# Sensitivity to microstimulation of somatosensory cortex distributed over multiple electrodes

**DOI:** 10.3389/fnsys.2015.00047

**Published:** 2015-04-10

**Authors:** Sungshin Kim, Thierri Callier, Gregg A. Tabot, Francesco V. Tenore, Sliman J. Bensmaia

**Affiliations:** ^1^Department of Organismal Biology and Anatomy, University of ChicagoChicago, IL, USA; ^2^Committee on Computational Neuroscience, University of ChicagoChicago, IL, USA; ^3^Research and Exploratory Development Department, Johns Hopkins University Applied Physics LaboratoryLaurel, MD, USA

**Keywords:** neuroprosthetics, intracortical microstimulation, discrimination task, detection performance, non-human primates

## Abstract

Meaningful and repeatable tactile sensations can be evoked by electrically stimulating primary somatosensory cortex. Intracortical microstimulation (ICMS) may thus be a viable approach to restore the sense of touch in individuals who have lost it, for example tetraplegic patients. One of the potential limitations of this approach, however, is that high levels of current can damage the neuronal tissue if the resulting current densities are too high. The limited range of safe ICMS amplitudes thus limits the dynamic range of ICMS-evoked sensations. One way to get around this limitation would be to distribute the ICMS over multiple electrodes in the hopes of intensifying the resulting percept without increasing the current density experienced by the neuronal tissue. Here, we test whether stimulating through multiple electrodes is a viable solution to increase the dynamic range of ICMS-elicited sensations without increasing the peak current density. To this end, we compare the ability of non-human primates to detect ICMS delivered through one vs. multiple electrodes. We also compare their ability to discriminate pulse trains differing in amplitude when these are delivered through one or more electrodes. We find that increasing the number of electrodes through which ICMS is delivered only has a marginal effect on detectability or discriminability despite the fact that 2–4 times more current is delivered overall. Furthermore, the impact of multielectrode stimulation (or lack thereof) is found whether pulses are delivered synchronously or asynchronously, whether the leading phase of the pulses is cathodic or anodic, and regardless of the spatial configuration of the electrode groups.

## Introduction

One approach to restoring sensorimotor function to patients with upper spinal cord injury consists of measuring signals from motor areas of their brains to control anthropomorphic robotic arms (Hochberg et al., 2012; Collinger et al., 2013). However, our ability to use our limbs relies heavily on somatosensory signals, which convey information about the consequences of our movements and about the objects with which we interact. With this in mind, it is necessary not only to re-establish the ability to send commands to the limb but also to restore the ability to receive sensory signals back from the limb. One strategy to restore somatosensation consists of electrically stimulating neurons in somatosensory cortex through chronically implanted electrode arrays in the hopes of eliciting meaningful tactile and proprioceptive sensations (London et al., 2008; O’Doherty et al., 2011; Berg et al., 2013; Tabot et al., 2013b, 2014; Thomson et al., 2013; Bensmaia and Miller, 2014; Dadarlat et al., 2014). One limitation of intracortical microstimulation (ICMS) is that high levels of current can damage neuronal tissue if the resulting current densities are too high. However, ICMS has been found to have a negligible effect on tissue over a range of current densities (up to about 1.0 mC/cm^2^; Rajan et al., unpublished observations), unless it is applied continuously for long periods of time (McCreery et al., 2010). One strategy to expand the dynamic range of elicited sensations without increasing the current density experienced by any one population of neurons, and thus to avoid damaging the brain, is to distribute the injected current over multiple electrodes (Zaaimi et al., 2013). That way, we might be able to achieve a wider dynamic range of sensations without subjecting neurons to higher peak current densities.

**FIGURE 1:**
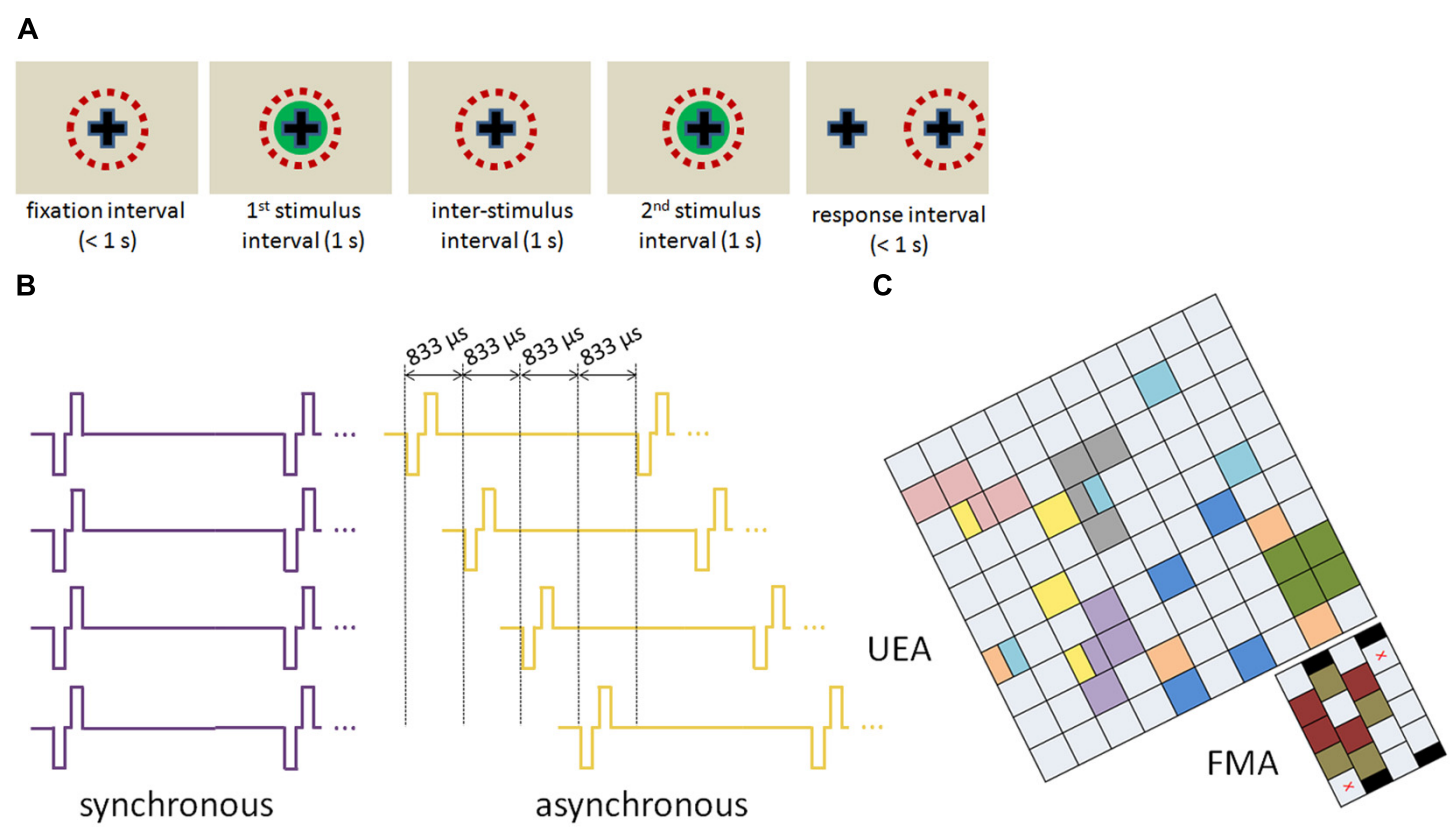
Experimental design. **(A)** Structure of a trial. The red dashed circle denotes the animal’s direction of gaze; on this example trial, the animal responded right. **(B)** Timing of synchronous and asynchronous ICMS for a quad of electrodes. **(C)** Spatial configuration of electrode quads from one monkey. Each square represents an electrode; electrodes that share a color were part of a quad (some electrodes were used in two quads).

To investigate this possibility, we had two non-human primates perform detection or discrimination tasks in a two-alternative forced choice paradigm (**Figure 1A**) to probe their sensitivity to ICMS delivered through one or more electrodes. Electrodes in each group were chosen such that their receptive fields were largely overlapping to ensure that the sensations evoked resulted in tactile sensations that were localized to a single location on the skin (cf. Tabot et al., 2013b). We wished to determine the degree to which stimulation through multiple electrodes (1) reduces the minimum amplitude required to achieve a percept (the absolute threshold) and (2) increases the number of discriminable amplitude increments [just noticeable differences (JNDs)] that can be achieved between absolute threshold and the maximum current per electrode (100 μA).

To these ends, we first investigated whether animals could better detect pulse trains delivered simultaneously through multiple electrodes (2 or 4) than they could the same pulse trains delivered through a single electrode. We also compared the animals’ sensitivity to multi-electrode stimulation when pulses were delivered synchronously or asynchronously within each stimulus cycle (**Figure 1B**). Moreover, we probed the effect on sensitivity of polarity (that is, whether the leading phase is cathodic or anodic) and of the spatial configuration of the electrodes on the array (**Figure 1C**). Finally, we investigated how multi-electrode stimulation affects the discriminability of ICMS pulse trains that differ in amplitude. We conclude that multi-electrode stimulation only provides a modest improvement in the dynamic range and does not justify its energetic cost.

## Results and Discussion

### Effect of Multi-Electrode Stimulation on Detectability

**FIGURE 2:**
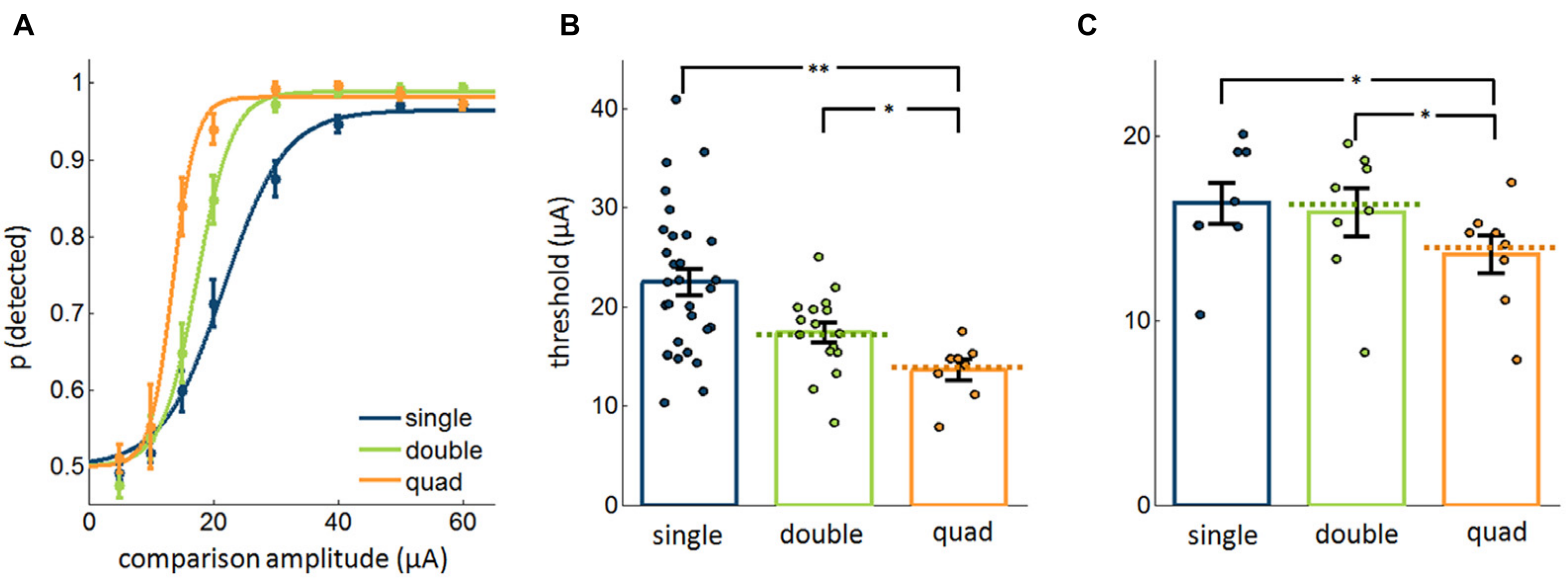
Detectability of ICMS delivered through varying number of electrodes (20,577 trials from 54 electrodes). In these experiments, pulses occurred synchronously (**Figure 1B**, left). **(A)** Psychometric functions, averaged across single electrodes, electrode pairs, and electrode quads. **(B)** Measured and theoretically calculated thresholds. The bars and error bars denote the mean and SEs, respectively, and each point corresponds to a different single electrode, electrode pair, or quad. The dashed lines denote the expected threshold assuming that each electrode independently contributes to detectability. **(C)** Comparison of the most sensitive single electrode, best pair of electrodes, and the quad with pulses delivered synchronously in the multi-electrode conditions (9,520 trials from 24 electrodes). Error bars denote the SE of the mean. ^∗^*p* < 0.05, ^∗∗^*p* < 0.01.

We compared detection performance for ICMS pulse trains delivered through 1, 2, or 4 electrodes with cathodic phase-first current pulses delivered synchronously across electrodes (**Figure 1B**, left). In these experiments, two and four times as much current was delivered in the double and quad conditions as was delivered in the single electrode condition, respectively. We found that the absolute threshold – defined as the ICMS amplitude that yielded 75% detection performance – decreased as the number of stimulated electrodes increased (**Figure 2A,B**; Kruskal–Wallis test, χ^2^_(2,51)_ = 14.8, *p* < 0.001), as might be expected given that more current was delivered to the brain (see Ghose and Maunsell, 2012; Zaaimi et al., 2013). A *post hoc* analysis revealed that, while single-electrode thresholds were not significantly different from electrode-pair thresholds (rank-sum test, Bonferroni corrected, *p* = 0.07), single thresholds were significantly higher than quad thresholds (*p* = 0.003), and double thresholds were significantly higher than quad thresholds (*p* = 0.04). Furthermore, thresholds measured in the multi-electrode conditions closely matched theoretical predictions based on the assumption that each electrode exerts an independent effect on detectability (signed rank test, *p* > 0.5, dashed lines in **Figure 2B**).

Next, we wished to assess the extent to which stimulation through multiple electrodes improves detectability beyond that achieved through stimulation of the most sensitive electrode. To this end, we compared performance with four electrodes to that with the best (most sensitive) electrode and with the best pair of electrodes in the quad. We found that, while not completely eliminated, the apparent advantage of multi-electrode stimulation was substantially reduced. Indeed, while detection performance remained significantly different between single electrodes and multiple electrodes (Friedman test, χ^2^_(2,14)_ = 9.25, *p* < 0.01, **Figure 2C**), the mean difference in threshold between groups was less than 3 μA (mean ± SEM: single vs. double: 0.50 ± 0.50 μA, single vs. quad: 2.76 ± 1.02 μA, double vs. quad: 2.26 ± 0.82 μA, mean ± SEM), representing a decrease of less than 10%. Importantly, the detectability of subthreshold stimuli (5 and 10 μA) did not improve significantly with multiple electrodes (Friedman test, χ^2^_(2,30)_ = 1.94, *p* = 0.4), which stands in contrast with previous findings (Zaaimi et al., 2013).

### Synchronous vs. Asynchronous Stimulation

**FIGURE 3:**
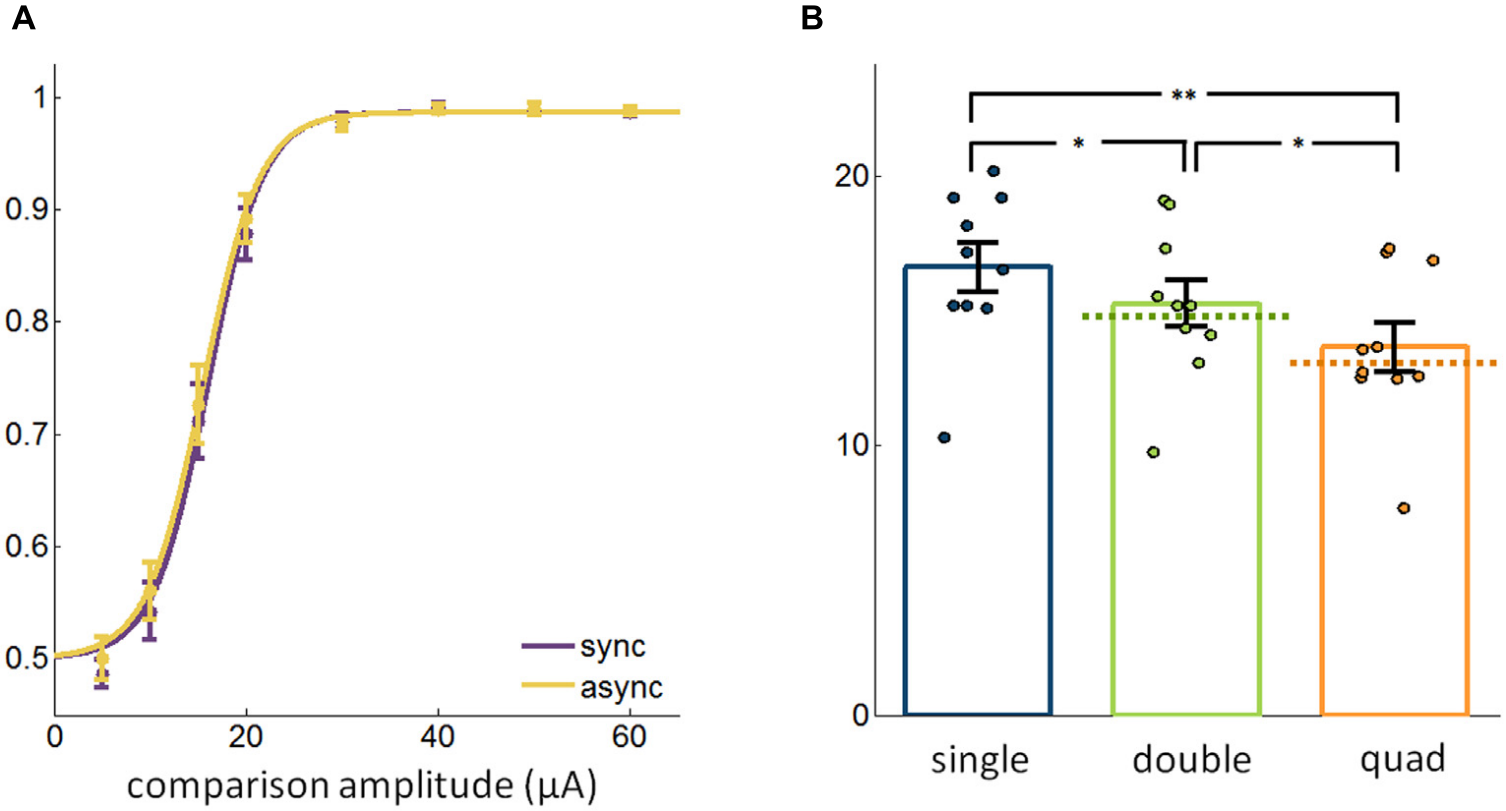
**(A)** Detection performance with synchronous and asynchronous stimulation (13,202 trials from 48 electrodes). **(B)** Comparison of the most sensitive single electrode, best pair of electrodes, and the quad with pulses delivered asynchronously in the multi-electrode conditions (19,400 trials from 30 electrodes). Error bars denote the SE of the mean. ^∗^*p* < 0.05, ^∗∗^*p* < 0.01.

In the multi-electrode conditions described above, electrical pulses were delivered synchronously through different electrodes. We wished to determine whether the effect of multi-electrode stimulation on sensitivity might be different when pulses are staggered rather than synchronous. To this end, we repeated the experiments described above, but interleaved trials in which pulses were delivered synchronously across electrodes with trials in which pulses were staggered (**Figure 1B**). First, we found that synchrony did not have a significant overall effect on thresholds [paired *t*-test, *t*_(23)_ = 1.03, *p* = 0.31] (**Figure 3A**), consistent with previous findings in optogenetic experiments with mice (Histed and Maunsell, 2014). Second, asynchronous multi-electrode ICMS had a similar effect on detectability as did its synchronous counterpart, with thresholds decreasing with more electrodes (Friedman test, χ^2^_(2,18)_ = 14.6, *p* < 0.001); a *post hoc* analysis revealed significant differences across all three groups (signed rank test, Bonferroni corrected, *p* < 0.05; **Figure 3B**). Additionally, as was the case with synchronous stimulation, measured thresholds were indistinguishable from theoretically estimated thresholds assuming independent contributions of each electrode to detection performance (signed rank test, double: *p* = 0.063, quad: *p* > 0.5). Again, the mean difference in threshold was small (single vs. double: 1.35 ± 0.32 μA, single vs. quad: 2.96 ± 0.43 μA, double vs. quad: 1.61 ± 0.38 μA) and the detectability of subthreshold stimuli did not improve significantly (Friedman test, χ^2^_(2,38)_ = 1.85, *p* = 0.40).

### Effect of Pulse Polarity on Detectability

**FIGURE 4:**
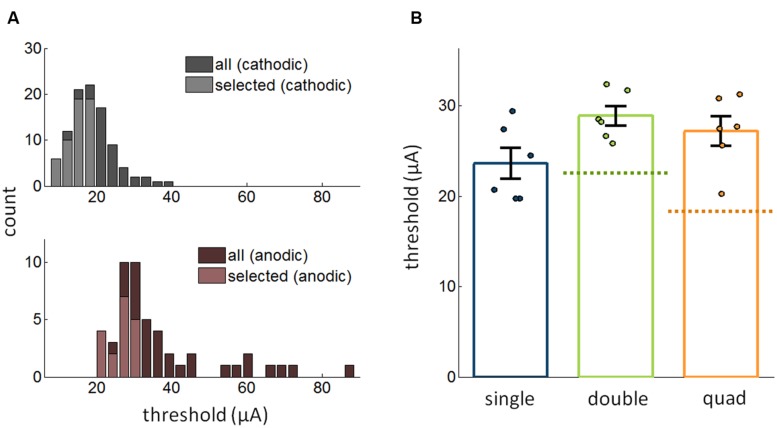
**(A)** Distribution of thresholds for anodic phase leading and cathodic phase leading ICMS (56,021 trials from 97 electrodes). The distribution of thresholds for the best electrodes is shown in the lighter hue. **(B)** Comparison of the most sensitive single electrode, best pair of electrodes, and the quad with pulses in the multi-electrode conditions with anodic phase leading (12,661 trials from 18 electrodes). Error bars denote the SE of the mean.

Next, we investigated whether changing the polarity of the pulses might modulate how stimulation through multiple electrodes affects detectability. That is, we compared the effect of multi-electrode stimulation when the leading phase was anodic to that when leading phase was cathodic. First, as has been previously shown (Ranck, 1975; Schmidt et al., 1996; Koivuniemi and Otto, 2011), detection thresholds were significantly higher for anodic-first pulses than for cathodic-first pulses [**Figure 4A**; *t*-test: *t*_(70)_ = 12.2, *p* < 10^-18^]. As was the case for cathodic-first pulses, thresholds for anodic-first stimulation decreased as the number of electrodes increased (Kruskal–Wallis test, χ^2^_(2,45)_ = 6.68, *p* = 0.036). However, the increase in sensitivity with increasing number of electrodes was eliminated when the best single electrode and the best pair were compared to the quad [Friedman test, χ^2^_(2,10)_ = 2.33, *p* = 0.31, single/double/quad: 23.6 ± 1.70 μA/28.9 ± 1.08 μA/27.2 ± 1.64 μA; **Figure 4B**]. Thus, results using multi-electrode ICMS with anodic phase leading did not conform with theoretical predictions based on the assumption of independence.

### Effect of Electrode Spacing on Detectability

Electrodes that formed each quad were selected to have largely overlapping receptive fields. In some cases the electrodes were physically adjacent, but in others they were not (**Figure 1C**). We wished to assess whether the spatial configuration of the electrode groups might impact how stimulation through these is combined to culminate in a behavioral outcome. Using ICMS with cathodic phase leading, we found that spatial configuration had no impact on sensitivity to multi-electrode stimulation: threshold decreased as the number of electrodes increased, whether these were adjacent (Friedman test, χ^2^_(2,18)_ = 18.2,* p* < 0.001) or not (χ^2^_(2,14)_ = 6.75,* p* = 0.03). In both conditions (adjacent vs. non-adjacent), observed thresholds were consistent with the assumption of independence; that is, these were not significantly different from predicted ones regardless of spatial separation (signed rank test,* p* > 0.1). The effect of separation was similar whether stimulation was presented synchronously or asynchronously, as might be expected from **Figure 3**.

### Multi-Electrode Stimulation for Discrimination

**FIGURE 5:**
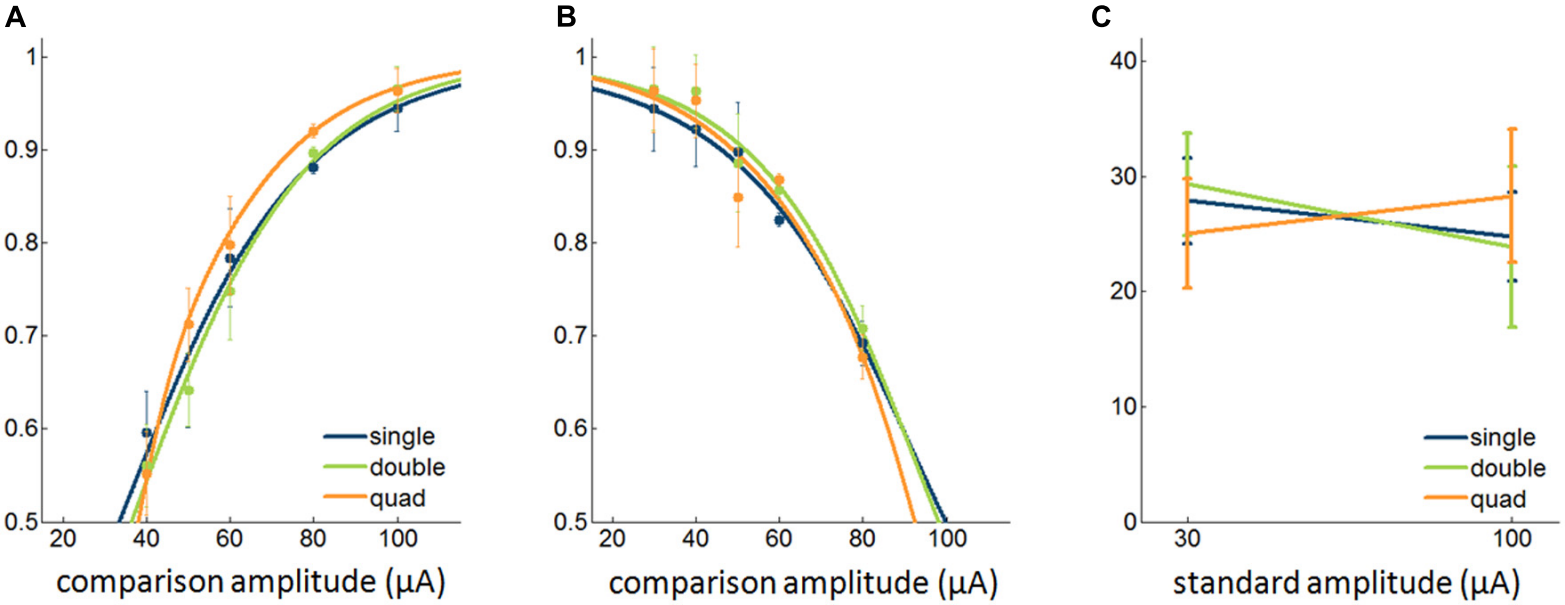
Effect of multi-electrode stimulation on the discriminability of ICMS. **(A,B)** Psychometric functions with a 30-μA and 100-μA standard, respectively (9,280 trials from 12 sets of electrodes). **(C)** JNDs with different standard amplitudes. Error bars denote the SEM.

Based on results from the detection experiments, we concluded that the detectability of ICMS improves only slightly when stimulation is delivered through multiple electrodes despite the fact that more current is injected. Next, we wished to examine whether the discriminability of ICMS pulse trains differing in amplitude increased when these were delivered through multiple electrodes simultaneously. To this end, we had animals discriminate ICMS that differed in amplitude, with stimuli delivered through single electrodes, pairs, or quads of electrodes. That is, we compared discrimination performance when both stimuli were presented through one, two, or four electrodes. In these experiments, pulses were anodic phase leading. We found that there was no significant difference in discrimination performance across the three conditions with either the 30-μA standard [Friedman test, χ^2^_(2,59)_ = 0.03, *p* = 0.99] or the 100-μA standard (χ^2^_(2,59)_ = 1.12,* p* = 0.57; **Figure 5**).

### Implications for Neuroprosthetics

The results of our detection experiments are consistent with the hypothesis that each electrode exerts an independent effect on sensitivity, except perhaps when the anodic phase leads, where no effect was observed (though the sample was relatively small for that condition). As such, the advantage of multi-electrode stimulation is relatively modest when compared to the “best” electrode, with four electrodes yielding a mean decrease in threshold of less than 10%. There was no effect of multi-electrode stimulation on discrimination, likely reflecting the fact that discrimination is generally less sensitive to stimulus parameters than is detection. The lack of effect on discrimination performance was probably exacerbated by the fact that these experiments were carried out with anodic phase leading.

At first glance, our results seem generally inconsistent with those reported in a previous study (Zaaimi et al., 2013), in which a supra-additive effect of multi-electrode stimulation on sensitivity was reported. Furthermore, in this previous study, the synergistic effects of multi-electrode stimulation were observed even for subthreshold stimuli, which was not the case here. However, in that study, the supra-additive effect was strongest when five or more electrodes were simultaneously stimulated, so perhaps we did not stimulate a sufficient number of electrodes in the present study to observe it. The discrepancy regarding the effect of multi-electrode stimulation on subthreshold stimuli may be attributable to differences in somatosensory areas that were stimulated (areas 3b/1 vs. area 2), in the relevant sensory modalities (tactile vs. proprioceptive), or in the behavioral protocols (one stimulus interval vs. two, 360-ms vs. 1000-ms stimulus duration, etc.).

Whether pulses were delivered synchronously or asynchronously did not affect their detectability, a result that is consistent with previous findings in mice using optogenetic stimulation (Histed and Maunsell, 2014). At peri-threshold, amplitudes, the current may spread to a volume with a radius of 200–300 μm or less (Stoney et al., 1968; Tehovnik et al., 2006; Zaaimi et al., 2013), so the different electrodes may have activated mostly non-overlapping populations of neurons. Even if the fields do interact, it may be that both synchronous and asynchronous stimulation have their respective advantages: with synchronous stimulation, the fields interact, allowing intervening neurons to experience stronger stimulation, thereby increasing their probability of firing; with asynchronous stimulation, neurons experience more continuous stimulation, thereby increasing their probability of firing; the two effects may then be approximately equivalent.

Given its limited effect on sensitivity, stimulation through multiple electrodes is not a very promising way to extend the dynamic range of sensations achievable through ICMS, at least for artificial touch and given current electrode technologies. Indeed, stimulating through the “best” electrode yields nearly equivalent results and requires a fraction of the current as does stimulating through four electrodes. One might argue that, given an independent contribution of each electrode, performance should improve as the number of stimulated electrodes increases. However, more electrodes will likely evoke more diffuse percepts (cf. Tabot et al., 2013b), so gains in dynamic range will be at the expense of spatial localization. On the other hand, for more distributed representations, such as proprioceptive ones, multi-electrode stimulation may be more practical (cf. Zaaimi et al., 2013). As technology develops, and implanted electrodes get closer together, the multi-electrode approach may be viable for touch as well. In the meantime, manipulations of phase width, pulse frequency, and pulse train duration may be more promising avenues to extend the dynamic range (Tabot et al., 2013a; Kim et al., 2014).

## Materials and Methods

### Animals

Procedures were approved by the University of Chicago Animal Care and Use Committee. Each of two male Rhesus macaques (6 years of age, around 10 kg in weight) was implanted with three electrode arrays: one Utah electrode array (UEA; Blackrock Microsystems, Inc., Salt Lake City, UT, USA) in the hand representation of areas 1 and 2 in the right hemisphere, flanked by two FMAs (Microprobes for Life Science, Gaithersburg, MD, USA) in area 3b (For more detail, see Berg et al., 2013; Tabot et al., 2013b). We mapped the receptive field of each electrode by identifying which areas of skin evoked multiunit activity (monitored through speakers).

### Experimental Design

Each trial consisted of two sequentially presented stimulus intervals, one (detection) or both (discrimination) of which contained a stimulus (**Figure 1A**). In the detection task, the animal indicated which of the two stimulus intervals contained the stimulus; in the discrimination task, the animal indicated which of the two intervals contained the more intense stimulus. In both tasks, the animals responded by making a saccade to one of two visual targets. Animals were first trained on these tasks with mechanical indentations delivered to their skin until their performance leveled off. Mechanical stimuli were then replaced with ICMS; importantly, the animals performed at a high level on the very first block of ICMS, suggesting that the ICMS detection and discrimination were very similar to their mechanical counterparts.

Intracortical microstimulation consisted of 1-s long trains of symmetric biphasic pulses with a phase duration of 200 μs, an interphase interval of 53 μs, and a frequency of 300 Hz (**Figure 1B**). In the multi-electrode conditions, ICMS was either delivered synchronously (with all pulses in a given cycle occurring simultaneously) or asynchronously, such that pulses were evenly distributed throughout the cycle (that is, with an interpulse interval of 1667 μs for pairs and 833 μs for quads of electrodes; **Figure 1B**). In each experimental block, trials with a single electrode were interleaved with trials with pairs or quads of electrodes. In all cases, all of the electrodes in a quad had largely overlapping receptive fields on the palmar surface of the hand. Each quad was broken down into pairs, so that we could compare performance with quad stimulation to that with stimulation through electrode pairs or through single electrodes using repeated measures statistics.

In the detection experiments, ICMS amplitude was 5, 10, 15, 20, 30, 40, 50, or 80 μA and varied from trial to trial in pseudorandom order. In the discrimination experiments, the same number of electrodes was used in both intervals of each trial; the animal was comparing ICMS delivered through one, two, or four electrodes. On each trial, the amplitude of the standard stimulus was 30 or 100 μA. The 30-μA standard was paired with comparison stimuli at 40, 50, 60, 80, or 100 μA. The 100-μA standard was paired with comparison stimuli at 30, 40, 50, 60, or 80 μA. The standard stimulus was presented in either the first or the second interval and trials with both standards were interleaved so the animal would have to pay attention to both intervals to perform the task correctly.

### Analysis

In the detection task, we estimated the detection threshold as the stimulus amplitude that yielded a performance of 75% correct. Similarly, in the discrimination task, we estimated the JND as the difference between comparison and standard amplitude that yielded a performance of 75% correct. Thresholds and JNDs were estimated using a standard sigmoid function. To compare sensitivity across conditions, we used parametric tests (e.g., *t*-tests) or non-parametric ones (e.g., Kruskal–Wallis test, Friedman test and signed rank test) depending on the sample size and variance of the data.

We also wished to quantify the expected performance if we assume that each electrode independently contributes to perception (cf. Zaaimi et al., 2013):

$$\begin{array}{c} P_{D}=1-\left(1-P_{S1}\right)\left(1-P_{S2}\right)\\ {\begin{array}{c} P \end{array}}_{Q}=1-\left(1-P_{S1}\right)\left(1-P_{S2}\right)(1-P_{S3})\left(1-P_{S4}\right) \end{array}\,\;\;\;\;\;\;\;\;\;\;\;\;\;\;\;\;\;\;\;\;\;\;\left(1\right)$$

Where *P_D_* and *P_Q_* indicate the probability of detection with pairs and quads of electrodes, respectively, and *P_S1_*, *P_S2_*, *P_S3_*, and *P_S4_* denote the detection probability with each of the individual electrodes in the pair or quad. The proportion correct observed in the detection task, *P_obs_*, is related to the probability of detection, *P_det_*, as follows:

$$P_{obs}=P_{\det}+0.5\left(1-P_{\det}\right)\,\;\;\;\;\;\left(2\right)$$

So the probability of detection is given by P_det_ = 2P_obs_ -1. We computed *P_det_* for each electrode and plugged the resulting value into Equation 1 to obtain the theoretical detection probability for double and quad electrodes. Finally, we used Equation 2 to convert the probabilities back to task performance metrics.
